# Supplementary material for: Eliminating mother-to-child transmission of HIV in Tanzania calls for efforts to address factors associated with a low confirmatory test
Source: PLOS Glob Public Health. 2023 Mar 13;3(3):e0001218. doi: 10.1371/journal.pgph.0001218 (PMC10021727; doi:10.1371/journal.pgph.0001218)
Supplement: Attachment — Submitted filename: Barakas Response to Reviewers.doc [file pgph.0001218.s001.doc]

**Manuscript Title: Eliminating mother-to-child transmission of HIV in Tanzania calls for efforts to address factors associated with a low confirmatory test**

**Corresponding Author: Baraka Malaki Morris**

**Response to Reviewers’ comments on Manuscript (PGPH-D-22-01598)**

| **Reviewer Name/#** | **Comment #** | **Comment** | **Response(s)** |
| --- | --- | --- | --- |
| Journal Requirements | 1. | Please amend your detailed Financial Disclosure statement. This is published with the article. It must be completed in full sentences and contain the exact wording you wish to publish.   a. Please clarify all sources of funding (financial or material support) for your study. List the grants (with grant number) or organizations (with URL) that supported your study, including funding received from your institution. b. State the initials, alongside each funding source, of each author to receive each grant. | This research project was part of the Afya Bora Consortium (ABC) Fellowship in Global Health Leadership 2017/2018 cohort. The ABC fellowship was funded by the U.S. President’s Emergency Plan for AIDS Relief (PEPFAR) through funding to the University of Washington’s International AIDS Education and Training Center (IAETC) under cooperative agreement U91 HA06801 from the Health Resources and Services Administration (HRSA) Global HIV/AIDS Bureau. The authors received no specific funding for this research work; however, the ABC fellowship provided a monthly stipend, research sites, and site mentors. The views presented here do not necessarily reflect the views of these funding agencies. |
|  | 2. | Please indicate by return email the full and correct funding information for your study and confirm the order in which funding contributions should appear. |  |
| Reviewer #1 | 1. | The research aim for the study was well-defined, but it seems to focus on aspects related to confirmatory HIV testing. The author should discuss the treatment adherence level of PMTCT elaborately among mother-baby. | These data focused on the EID and PMTCT. All infants who were diagnosed with HIV were referred to care and treatment clinics (CTC). However, for those mothers of babies who tested HIV negative during the DBS1, showing up for HIV confirmatory testing was one of the indicators for compliance with HIV treatment. |
|  | 2. | Literature Review: The various article is current and reflects issues found on PMTCT -option B+. Since PMTCT adherence varies, the Author should include current information on Option B mother-child adherence to treatment level. |  |
|  | 3. | Authors need to include how data will be protected after the study. | The PMTCT Unit of the Ministry of Health, Community Development, Gender, Elderly and Children (MoHCDGEC) Tanzania granted the corresponding author access to the data for this study only. Hence, the corresponding author has the responsibility to ensure that the data is well protected. To prevent unauthorized access, the data will be stored in a protected computer and the folder containing the data will be locked with a strong password. The permission to access the data will be given by the MoHCDGEC |
|  | 4. | This article would benefit from information on Antenatal data. The study objective implies monitoring and evaluating treatment among mother-baby enrolled in the option B approach of PMTCT, more discussion of this aspect would be beneficial. Most of the discussion focuses on factors related to option B compliance, with little discussion on the loss to follow up. | Lost to follow-up has been adequately discussed. Likewise, more citations on lost to follow-up have been added  Refer lines #291 - 323 |
| Reviewer #2 | 1. | Overall the study stated the early infant diagnosis, however the results of EID did not exist (information only whether DBS is collected or not), it will be better if adding information and comparing between those with EID Positive and EID negative results with other factors associated with it. | The results of DBS1-EID have been included in the result section. However, only results for 651(86%) mother-baby pairs were recorded. Moreover, in the data it was not clear whether they received the results within the first 2 months as it was recommended at that time. Studies have reported high delays in the central laboratory for DBS1-PCR analysis.  Refer Lines #215 – 218 |
|  | 2. | New WHO guidelines inform that in Page 5: WHO recommends that all HIV-exposed infants receive a virological test for HIV within six weeks of birth, followed by the initiation of ART for those who are infected; source : https://www.unicef.org/supply/sites/unicef.org.supply/files/2020-03/HIV-early-infant-diagnosis-viral-load-point- care-diagnostics-market-note.pdf | The recommendation have been included this update in the introduction and description of variables. However, the guideline used to develop the tool for data collection required babies to have BDS1 within their first 2 months of age.  Refer lines #69 – 75, 151 - 155 |
|  | 3. | Line 54: adding reference | Reference has been added |
|  | 4. | Line 208: deleted the last variable as already put in the column | Noted |
|  | 5. | Line 232-234: table need to be revise and add the information stated in line 216-218 | The table has been revised and the results for gravidity groups and gestation age groups has been added |
|  | 6. | Recommendation that needs to be added is based on WHO guidelines is early detection of infants using DBS within six weeks of birth and directly provided treatment once infected. | It is true that WHO recommend the first HIV testing (DBS1) to take place within the six week, even the Tanzania National Guidelines for the Management of HIV and AIDS of 2019 recommend the same. However, the two months reference was applicable during the data collection and hence the tool was developed based on that. This has been added in the discussion.  Refer line s #69 – 75, 151 - 155 |
|  | 7. | Below similar journal that can be cited to enrich the findings :   a. Article Source: Interventions to increase early infant diagnosis of HIV infection: A systematic review and meta-analysis Okusanya B, Kimaru LJ, Mantina N, Gerald LB, Pettygrove S, et al. (2022) Interventions to increase early infant diagnosis of HIV infection: A systematic review and meta-analysis. PLOS ONE 17(2): e0258863. <https://doi.org/10.1371 /journal.pone.0258863> b. Point-of-care testing can achieve same-day diagnosis for infants and rapid ART initiation: results from government programmes across six African countries https://doi.org/10.1002/jia2.25677 access through https://onlinelibrary.wiley.com/doi/10.1002/jia2.25677 | Thank you for these important citations, I have reviewed and included them to enrich the discussion  Refer lines #302 - 311 |
